# Improvement of tomato yield and quality using slow release NPK fertilizers prepared by carnauba wax emulsion, starch-based latex and hydrogel nanocomposite combination

**DOI:** 10.1038/s41598-023-38445-7

**Published:** 2023-07-10

**Authors:** Elaheh Motamedi, Marzieh Safari, Mehri Salimi

**Affiliations:** 1 Department of Nanotechnology, Agricultural Biotechnology Research Institute of Iran (ABRII), Agricultural Research, Education and Extension Organization (AREEO), Karaj, Iran; 2 Division of Plant and Soil Sciences, Davis College of Agriculture, Natural Resources and Design, West Virginia University, Morgantown, USA; 3 Soil Science Department, College of Agriculture and Natural Resources, University of Tehran, Karaj, Iran

**Keywords:** Biotechnology, Chemistry

## Abstract

The modern agriculture is working on introducing new generation of fertilizers that apt to slow down the nutrients release to be more in synchrony with plant’s need throughout growth season, enhance fertilizer performance, and decrease nutrient losses into the environment. The aim of this research was to develop an advanced NPK slow-release fertilizer (SRF) and investigate its effect on yield, nutritional and morphological responses of tomato plant (*Lycopersicon esculentum* Mill.) as a model crop. To this goal, three water-based bio-polymeric formulations including starch-*g*-poly (acrylic acid-*co*-acrylamide) nanocomposite hydrogel, starch-*g*-poly(styrene-*co*-butylacrylate) latex, and carnauba wax emulsion were synthesized and used for production of NPK-SRF samples. Different samples of coated fertilizers (urea, potassium sulfate, and superphosphate granules) were prepared using different ratios of latex and wax emulsion, and for phosphorus and potash (R-treatment). Moreover, some of coated fertilizers (15 and 30 wt.%) was replaced with nanocomposite hydrogel containing fertilizers, named D and H treatments, respectively. The effect of SRF samples were compared with commercial fertilizers (NPK treatment) and a commercial SRF (T treatment), on the growth of tomato in the greenhouse, at two different levels (100 and 60). The efficiency of all the synthesized formulations were higher than NPK and T treatments, and among them, H100 significantly improved the morphological and physiological characteristics of tomato. For instance, amount of residual elements (nitrogen, phosphorus and potassium) as well as micro elements of calcium, iron and zinc in tomato cultivation bed and accordingly the uptake of these elements in the roots, aerial parts and fruits were increased in the R, H, and D treatments. The highest yield (1671.54 g), highest agricultural agronomy efficiency of fertilizer, and the highest dry matter percentage (9.52%) were obtained in H100. The highest amount of lycopene, antioxidant capacity and vitamin C was also observed in H100. Nitrate accumulation in tomato fruit in the synthesized SRF samples were decreased significantly compared to NPK100, and the lowest amount was observed in H100, which was 55.24% less than NPK100. Accordingly, it is suggested that combination of natural-based nanocomposite hydrogels along with coating latexes and wax emulsions can be a successful method to synthesize efficient NPK-SRF formulations for improvement of crop growth and quality.

## Introduction

Most conventional fertilizers comprised macronutrients of nitrogen (N), phosphorus (P) and potassium (K) which have low use efficiencies since only 50–60% of N and K, and 10–25% of P could be efficiently absorbed by the crops^1–3^, and the rest discharge from the system through volatilization, run-off or leaching resulting in environmental pollution^4–6^. To tackle such disadvantages of conventional nutrients, slow release fertilizers (SRFs) were received many interest not only to provide greater nutrients bioavailability but also to augment the reaction rates in plant cells, correspondingly improve plant quality via increasing the protein, oil, and sugar contents^3,7,8^. Three main strategies for preparation of SRFs are: low-solubility nutrients, coated nutrients, and matrix/entrapped nutrients^2,4,9,10^. Superabsorbent hydrogels (SH) are one of the most explored polymers utilized for SRF production because they can absorb water and nutrients simultaneously, hold them within structure tightly, hence release them slowly^9^. From the viewpoint of coated nutrients, common polymeric coating materials are included polyurethane, polysulfone, polyacrylonitrile, polyolefin, polyvinyl chloride and cellulose acetate^2^. Commercial SRFs mostly used synthetic polymer coatings of polyolefin, polyvinylidene chloride and so on, which cannot degrade easily, consequently accumulate overtime. In addition, most SH-based synthetic polymers including poly(acrylic acid), poly(acrylamide) and copolymer are non-biodegradable that contribute to environmental pollution^9^. Therefore, the research focuses has been relocated to the development of more eco-friendly, biodegradable, and cost effective materials from abundant/renewable resources^9^. In this regard, natural polymers including starch, chitosan, lignin, alginate, and cellulose could act as permeable or impermeable membranes with tiny pores to release nutrients slowly^6^. Among them, starch is a renewable natural polysaccharide, which exhibits great potentials due to its biodegradability, availability, and ecofriendly features^11^. It could significantly improves the water holding capacity of the soils owing to its hydrophilic nature, network structure, and its ability to form strong hydrogen bonds with water molecules^12^. However, the application of native starches without any modifications is limited since they have least compatibility with hydrophobic polymers and do not have adequate mechanical strength and viscosity required for coating^2,9,13^. Hence, starch is being blended or copolymerized with different natural and synthetic polymers including rubber, lignin and ethyl cellulose, polyvinyl alcohol, poly(l-lactide), poly (acrylic acid-*co*-acrylamide), poly(vinyl acetate-*co*-butyl acrylate), and poly(acrylic acid) in different studies^12,14–16^. More specifically, there are various reports on the efficiency of starches with different ratios of amylose and amylopectin^17^, starch grafted onto a poly(acrylic acid-*co*-acrylamide) [starch/P(AAc-*co*-AAm)] hydrogel^18^, encapsulated urea with an internal cross-linked layer of starch and an external coating composed of acrylic acid and acrylamide^19^, polymeric matrix of starch-polyvinyl alcohol (PVA) reinforced with carbon nanofibers^20^, and the composite of chitosan/starch-hydrogel to prepare proficient SRF formulations^21^.

Apart from bio-based hydrogels and polymeric coatings used for SRF production, natural waxes which are the mixture of hydrophobic materials derived from plant cuticles or insects secretion, could be effectively utilized for coating fertilizers^22–25^. Among various industrial natural waxes, carnauba wax (CW, provided from leaves of the *Copernica prunifera*) with strong hydrophobic character and proper adhesion/friction resistance seems as an excellent candidate for synthesis of SRFs^26–29^. Recently, application of CW in modification of polyacrylate polymer to prepare double-layer coated urea were reported in which CW could noticeably improve the coating hydrophobicity and water-tightness resulting in SRF sample with long controlled release (70 days)^26^. Although commercial SRFs have shown the potentials for more nutrient efficiency, and reducing the environmental impacts of leaching nutrients but more innovations should be applied in production of natural-based SRF materials. Such advanced SRFs would be safer and more biodegradable, have the maximum nutrient content, and adequate release control, that could decrease the production costs. Inspiring our earlier research works on the synthesis of starch-based waterborne latexes and hydrogels for preparation of slow release urea^30,31^, it could be hypothesized that combining these two different methods (loading nutrient on hydrogel and coating nutrient with latex and/or wax emulsions) would result in more efficient SRF samples. Hence, in this study, the first aim was investigation the effect of the advanced NPK-SRF samples which were prepared through combination of different formulations: (starch-*g*-poly (acrylic acid-*co*-acrylamide) nanocomposite hydrogel, starch-*g*-poly(styrene-*co*-butylacrylate) latex, and carnauba wax emulsion. The second aim was evaluating the performances of the as-produced compound SRF samples on the yield, nutritional and morphological responses of tomato crop to confirm their potential applications and proof our hypothesis. To these goals, the efficiencies of the synthesized SRF formulations via different ratios of hydrogel, latex and wax emulsions were compared and contrasted with the traditional fertilizers (urea, potassium sulfate, and single superphosphate) and a commercial-SRF sample, on tomato production in the greenhouse experiment and the results confirmed the potency of these advanced and innovative SRFs in improving the crop yield and quality. Finally, this should be mentioned that since the research has been carried out in an inert substrate, the results could not be generalized for soil crops in the fields, and additional research studies in various soils (with different textures/pHs), for different field crops and in the repeated seasons are required for practical development of these samples that all are the ongoing and/or future research plans of this research team.

## Materials and methods

### Chemicals

Potassium permanganate (KMnO_4_), corn starch, ammonium persulfate (APS, (NH_4_)_2_S_2_O_8_), styrene (C_6_H_5_CH=CH_2_), butyl acrylate (BA, C_4_H_9_O_2_CCH=CH_2_), Tween 80 (C_64_H_124_O_26_), oleic acid (C_18_H_34_O_2_), acrylamide (AAm, CH_2_=CHC(O)NH_2_), acrylic acid (AA, CH_2_=CHCOOH), *N,N*-methylene bisacrylamide (MBA, CH_2_[NHCCH=CH_2_]_2_), ammonia solution (NH_4_OH, 30%), sulfuric acid (H_2_SO_4_, 98%), ethanol (C_2_H_5_OH, 95%), hydrochloric acid (HCl, 37%), hydrogen peroxide (H_2_O_2_, 30%) were purchased from Sigma–Aldrich. Carnauba wax flakes were bought from Pasargad Novin Co. Granular urea (U), single superphosphate (P) and potassium sulphate (K) were purchased from local market. The raw natural char (NC) was collected from Kuhbanan, Kerman, Iran. Other reagents were of analytical grade and obtained from Sigma–Aldrich Co..

### Synthesis of SRF samples

#### Loaded fertilizers in nanocomposite hydrogel

Firstly, natural char nanoparticles (NCNPs) were synthesized via chemical oxidation of raw NC which were reported in details in our earlier study^32^. Then, for preparation of the reinforced starch-based hydrogel nanocomposite (NCNPs/starch-g-poly(acrylic acid-co-acrylamide), 7.5 g starch was dispersed in distilled water (100 ml) using a mechanical stirrer. Next, NCNPs (75 mg) was separately suspended in distilled water through 15 min ultrasonication, added to the starch reaction flask and the mixture was stirred for 30 min. After that, 3.75 g of AAm, 28 ml of AA solution (60% neutralized with KOH), and 375 mg of cross-linker (MBA) were added to the mixture followed by addition of the initiator (0.75 g of APS). The mixture was stirred vigorously by a mechanical stirrer at 80 °C until preparation of a sticky gel which was coagulated in methanol, then washed and dried in an oven (60 °C)^30,32^. The as-prepared hydrogel nanocomposite was then utilized for loading of three NPK fertilizers. To this goal, urea, single superphosphate, and potassium sulfate were separately dissolved in water (10%w/v). Then, 100 ml of each solution was added to 2 g of hydrogel nanocomposite and allowed to swell and uniform gel was formed. The gels were dried in an oven overnight, at 40 °C, and the three loaded fertilizer in hydrogel nanocomposite structure were coded as U/Hydrogel, P/Hydrogel, and K/Hydrogel, for urea, phosphorous and potash fertilizers, respectively.

#### Synthesis of starch-based latex

Firstly, latexes were prepared via radical polymerization of styrene and BA monomers on starch backbone in the presence of emulsifier. 5 g starch was dispersed in distilled water (100 ml) using a mechanical stirrer and then the nonionic emulsifier (tween 80, 0.4 g in 20 ml distilled water) was added to the reaction flask and the mixture was vigorously stirred for 10 min. Next, styrene (15 ml) and BA (15 ml) were added to the mixture and then initiator (APS, 0.2 g in 20 ml distilled water) was added. After 20 min stirring, the reaction temperature was raised up to 85 °C, and the reaction content was mixed at this temperature for 3 h. Finally, the as-prepared polymer latex (coded as **F1**) was stored in closed containers after reaching the ambient temperature. To synthesize nanocomposite latex (coded as **F2**), 50 mg of NCNPs was suspended in 10 ml distilled water using an ultrasonic bath, and then added into the starch mixture before addition of monomers, the other steps was same as the aforesaid procedure for latex synthesis^31^.

#### Synthesis of carnauba wax emulsion

To prepare carnauba wax emulsion, a modified water-to-wax method^33^ was used where 5 g carnauba wax and 2 g oleic acid were heated in an oil bath (up to 80 °C) to melt and mix. After that, 1.5 g paraffin was added to the reaction mixture followed by addition of 2 ml ammonia solution which turned the viscose syrup mixture color from honey-yellow to dark brown. Finally, 70 ml of hot water was gradually added to the reaction to produce a creamy emulsion. Then, the prepared emulsion (coded as **F3**) was cooled to ambient temperature under mechanical stirring, and stored in closed containers for further use.

#### Coating of urea, single superphosphate and potassium sulfate fertilizers

For fertilizer coating, fertilizer granules were separately poured into a rotary drum instrument. As presented in Table 1, the specific amounts of formulations 1 to 3 was sprayed on the surface of fertilizers. Spun at 60 rpm, and the drum contents were heated at 85 °C for around 20 min until a uniform coating was created on the fertilizer surfaces, and the water was completely evaporated.

**Table 1:** Coating of fertilizer granules with different ratios of coating formulations. *F1* starch-based latex, *F2* starch-based nanocomposite latex, *F3* carnauba wax emulsion.

Code	Urea (g)	F1 (ml)	F2 (ml)	F3 (ml)	Ratio 1:1 of F1 + F3 (ml)	Ratio 1:1 of F2 + F3 (ml)
U1	100	50	–	–	–	–
U2	100	25	–	–	–	–
U3	100	–	50	–	–	–
U4	100	–	25	–	–	–
U5	100	–	–	50	–	–
U6	100	–	–	25	–	–
U7	100	–	–	–	50	–
U8	100	–	–	–	25	–
U9	100	–	–	–	–	50
U10	100	–	–	–	–	25

Type of fertilizer	Code	Fertilizer weight (g)	F2 (ml)	Ratio 1:1 of F2 + F3 (ml)		
Potassium sulfate	K1	250	–	125		
	K2	250	–	75		
	K3	250	125	–		
Single superphosphate	P1	250	–	125		
	P2	250	–	75		
	P3	250	125	–		

In order to repeat the coating after the drying of the first coating layer on the fertilizers (specifically in case of urea granules), formulations were sprayed again on the coated urea granules. Urea fertilizer coated with different ratios of formulations 1 to 3 (Table 1) coded as **U1** to **U10**, and their double coat samples were coded as **U1-2** to **U10-2**, which coating conditions in the first and second runs were same. The coated single superphosphate samples coded from **P1** to **P3**, and coated potassium sulfate samples coded from **K1** to **K3** (Table 1). While the most used potash fertilizer in agriculture is potassium chloride (due to its lower cost), in this research potassium sulphate was selected as potash source due to its lower salt index and lower water solubility.

The amount of each element in the coated fertilizers could be estimated by measuring the weight of the fertilizer granules before and after coating process. Using this method, the average wight percent of coating layer was calculated as 9 w% of total weight of coated fertilizer. In case of single layer coated urea, this meant that 1 kg of coated urea contained 0.91 kg (910 mg) of urea plus 0.09 kg (90 mg) of coating. As urea contain 46% nitrogen, the N content of single layer coated urea contained 41.8% N, and double layer coated urea contained 37.7% of N. The P of coated single superphosphate (16% P_2_O_5_) could be similarly estimated as 14.5% P_2_O_5_ (16 × 0.91) and K content of coated potassium sulfate (50% K_2_O) was calculated 45.5% K_2_O (50 × 0.91).

### Greenhouse experiments

#### Treatments

The greenhouse experiments were carried out in pots containing 2 kg of the substrate [coco peat:perlite:sand in proportion of 1:1:1 (v:v:v)], from September 2020 to April 2021, at Agricultural Biotechnology Research Institute of Iran (ABRII). Cherry tomato (*Solanum lycopersicum* var. *cerasiforme*) seeds were purchased from online shop for agricultural products in Iran (https://shop.golbargepamchal.com), and employed for the cultivation in the pots. The efficiency of the as-synthesized SRF samples were compared and contrasted with a commercial SRF and traditional (uncoated) fertilizers, on growing cherry tomato plants. The effects of five fertilizer treatments including R (NPK coated with coating formulations), D and H (NPK coated with coating formulations + NPK loaded in nanocomposite hydrogel), T (commercial SRF: Seasol-PowerFeed-Tomato & vegetables), traditional NPK (without coating/load), at two levels (100% of the required fertilizer according to the soil test, and 60% of the recommended fertilizer), and Blank (no fertilizer), were evaluated with five replicate, within a randomized complete block design with a factorial arrangement (Table 2). The detailed amounts of different fertilizer codes utilized in each treatment (R, D and H), and detailed of fertilizer treatments in each pot details of treatments were summarized in Tables 2 and 3, respectively.

**Table 2:** The amounts of coated fertilizers (g) for the preparation of R, D and H fertilizer treatments at two different levels. **U1** to **U1:** urea fertilizer coated with different ratios of coating formulations; **U1-2** to **U10-2:** double coat urea granules with coating formulations (coating conditions in the first and second runs were same). **P1** to **P3:** coated single superphosphate granules with coating formulations, **K1** to **K3:** coated potassium sulfate granules with coating formulations; U/Hydrogel: loaded in nanocomposite hydrogel; P/Hydrogel: single superphosphate loaded in nanocomposite hydrogel; K/Hydrogel: potassium sulfate loaded in nanocomposite hydrogel. Details of preparation of each coded sample were summarized in Table 1.

Treatment	U2 + U4	U1 + U3 + U6	U7 + U10 + U1-2 + U2-2	U9 + U3-2 + U4-2	U6-2 + U8-2 + U10-2	U5-2 + U7-2 + U9-2	U/Hydrogel	
R(100)	0.4	0.4	0.8	1.2	0.75	0.4	–	
R(60)	0.24	0.24	0.48	0.72	0.45	0.24	–	
D(100)	0.34	0.33	0.8	1	0.66	0.33	0.6	
D(60)	0.2	0.21	0.4	0.63	0.4	0.21	0.35	
H(100)	0.28	0.27	0.56	0.84	0.54	0.27	1.3	
H(60)	0.18	0.18	0.36	0.51	0.33	0.18	0.7	

Treatment	K1	K2	K3	K/Hydrogel	P1	P2	P3	P/Hydrogel
R(100)	4	1.6	2.4	–	0.7	0.3	0.7	–
R(60)	2.4	1	1.5	–	0.5	0.5	0.2	–
D(100)	3.6	1.4	1.9	1.3	0.6	.025	0.6	0.2
D(60)	2.2	0.9	1.2	0.8	0.4	0.15	0.4	0.15
H(100)	2.8	1.2	1.7	32.3	0.5	0.2	0.5	0.25
H(60)	1.7	0.7	1	1.4	0.3	0.15	0.3	0.4

**Table 3:** Amounts of different fertilizer treatments in each pot.

Fertilizer	Urea		Single superphosphate		Potassium sulfate	
Treatment	Coated U (g)	U/Hydrogel (g)	Coated P (g)	P/Hydrogel (g)	Coated K (g)	K/Hydrogel (g)
R (100)	4	–	1.7	–	8	–
R (60)	2.4	–	1.2	–	5	–
D (100)	3.4	0.6	1.45	0.2	6.9	1.3
D (60)	2	0.4	0.95	0.15	4.3	0.8
H (100)	2.8	1.3	1.2	0.25	5.6	2.3
H (60)	1.7	0.7	0.75	0.4	3.4	1.4
T (100)	13.7 g of commercial fertilizer containing NPK elements					
T (60)	8.6 g of commercial fertilizer containing NPK elements					
NPK (100)	4		1.7		8	
NPK (60)	2.4		1.2		5	
Blank	–		–		–	

Coated U: urea coated with polymer latexes and wax emulsion; Coated P: single superphosphate coated with polymer latexes and wax emulsion; Coated K: potassium sulfate coated with polymer latexes and wax emulsion; U/Hydrogel: loaded in nanocomposite hydrogel; P/Hydrogel: single superphosphate loaded in nanocomposite hydrogel; K/Hydrogel: potassium sulfate loaded in nanocomposite hydrogel.

Plants cultivation in the pots started with placing cherry tomato seeds on the moist filter paper in Petri dishes for 24 h, four germinated seeds were planted in each pot. After emergence and the development of true leaves, thinning was done, and one seedling was left inside each pot. During the growing period, a relative humidity of 40% was considered, the greenhouse temperature was kept at 26/23 °C (day/night), there were 16/8 h light/dark cycles with light intensities between 14,000 and 15,000 lx. In order to maintain the soil moisture at 80% of the field capacity, irrigation was done when needed. Since the commercial SRF sample contained calcium and micronutrients (iron and zinc), these elements were added every two weeks to the pots of R, H, D, and NPK treatments with irrigation. The plants were harvested after the end of the fruiting period. All procedures were conducted in accordance with the guidelines.

#### Comparison the release rate of SRF samples in the cultivation substrate

In order to check the release rate of nitrogen, phosphorus and potassium from fertilizers at different times (5, 10, 20, 30, 40, 50, 60, 70, 80 and 90 days) for each fertilizer treatment, 10 pots were prepared. 100 g of substrate (a mixture of sand, coco peat and perlite with a ratio of 1:1:1) was distributed into each pot, and the specific amounts of fertilizer treatments (R, D, H, T and NPK) placed in the hand-made fabric bags and added to the pots (Table 4). After interval time, the bags containing the residual fertilizer withdrew and the amount of the nitrogen, phosphorus and potassium contents in the air-dried substrate were measured.

**Table 4:** Amounts used for different fertilizer treatments in each pot in order to check the release of nutrients.

Fertilizer	Urea		Single superphosphate		Potassium sulfate	
Treatment	Coated U (mg)	U/Hydrogel (mg)	Coated P (mg)	P/Hydrogel (mg)	Coated K (mg)	K/Hydrogel (mg)
R (100)	250	–	100	–	500	–
D (100)	210	40	85	15	425	75
H (100)	175	75	70	30	350	150
T (100)	850 mg of commercial SRF fertilizer					
NPK (100)	250		100		500	

Coated U: urea coated with polymer latexes and wax emulsion; Coated P: single superphosphate coated with polymer latexes and wax emulsion; Coated K: potassium sulfate coated with polymer latexes and wax emulsion; U/Hydrogel: loaded in nanocomposite hydrogel; P/Hydrogel: single superphosphate loaded in nanocomposite hydrogel; K/Hydrogel: potassium sulfate loaded in nanocomposite hydrogel.

#### Greenhouse measurements

Before harvesting, some plant characteristics including chlorophyll index and after harvesting stem length, fresh and dry weight of roots, number of fruits, fresh and dry weight of fruit, fruit size, as well as the amount of nitrogen, phosphorus, potassium, zinc, iron, calcium in the growing medium, root, steam and fruit, the amount of lycopene, vitamin C, antioxidant capacity in the fruit, nitrate concentration in the fruit, and agronomic use efficiency (AUE) of the applied fertilizer (Eq. 1) were determined.

1 $$AUE=\frac{YX-Y0}{Nf}$$

where YX is the yield in each fertilizer treatment (g), Y0 is the yield in control plants (without fertilizer application), and Nf is the consumed fertilizer (g).

Chlorophyll index was measured using a chlorophyll meter (SPAD-502Plus). For this purpose, three leaves from each plant and two points from each leaf were randomly measured and finally their average was recorded. Three sites on top, bottom, and between were examined using a vernier scale to measure stem diameter, and the average amount was noted. Fresh shoots weights were measured after plants were cut at the crown. To measure the root dry weight, the roots were carefully taken out of the pots, properly cleaned with water to eliminate dust, and then placed in the oven (70 °C) to determine their dry weights. To evaluate the performance, fruit were harvested over two months, with a time interval of one week, and the product of each plant was weighed separately with a digital scale and the plant production over time was calculated. To measure the dry weight of the fruit, ten fruits were randomly selected. After recording the fresh weight, the samples were placed in special paper envelopes and dried in an oven at 60 °C. After being completely sure of drying, their dry weight was recorded and the percentage of dry matter based on the number of produced fruits was calculated.

ICP analysis (ICP OES device SPECTRO ARCOS model) was used to measure the amount of phosphorus, potassium, zinc, iron, and calcium elements in fruit, root, shoot, and substrates. Calibration standards were prepared from 1000 mg/L of calcium, potassium, iron, zinc and phosphorous solutions daily. The dried samples were separately ground using agate mortar, and 1.0 g of each powdered sample were weighed and dissolved in a mixture of 3 mL of HNO_3_ (65% w/v) and 1 mL of H_2_O_2_ (35% w/v), at 100 ℃, for 2 h. Digests were then diluted appropriately before analysis. Blanks were subjected to the same procedure and the results were given as the average of at least three replicates^34^. The amount of total nitrogen in fruits, roots, shoot, and substrates was determined by the Kjeldahl method. Powdered samples were separately mixed with a proportional amount of a concentrated sulfuric acid in a Kjeldahl flask. The resulting mixtures were heated until clarification as CO_2_ evolves, and ammonium sulfate solution was produced. Then, the Kjeldahl flasks were attached to a water condenser and NaOH solution was added to turn ammonium ions into ammonia. The produced ammonia gas was trapped in a receiving boric acid solution and the ammonia concentration was determined by titration^35^. Nitrate in tomatoes was analyzed using a spectrophotometer at a wavelength of 412 nm^36^. 100 mg of each powdered sample was suspended in 10 mL deionized water, the suspensions were incubated at 45 ℃, for 1 h. Then, the samples were centrifuged to sediment tissue residues, and supernatants were decanted and saved for analysis. Aliquots (0.2 mL) were mixed thoroughly with 0.8 mL of 5% (w/v) salicylic acid in concentrated sulfuric acid. After 20 min at room temperature, 19 mL of NaOH (2 M) was added slowly, to raise the pH of samples above 12. Then, the samples were cooled to room temperature and their absorbances were determined at 412 nm^36^. To measure lycopene, vitamin C and antioxidant capacity, five fruits were randomly selected from each replication and the sample was grated and frozen in liquid nitrogen. The samples were kept in a freezer at −80 °C until analysis, then HPLC (Agilent 1100 Series) equipped with RI detector and Symmetry^®^ NH2 chromatography column was used for the measurements^37–39^.

### Statistical analysis of data

Statistical analysis of data was done using R software 4.1.1., graphs were drawn using Excel, and data comparison was done using Tukey test at the 5% level.

## Results and discussion

### Release rate of the as-produced vs. commercial fertilizers in the cultivation substrate

**Figure 1:**
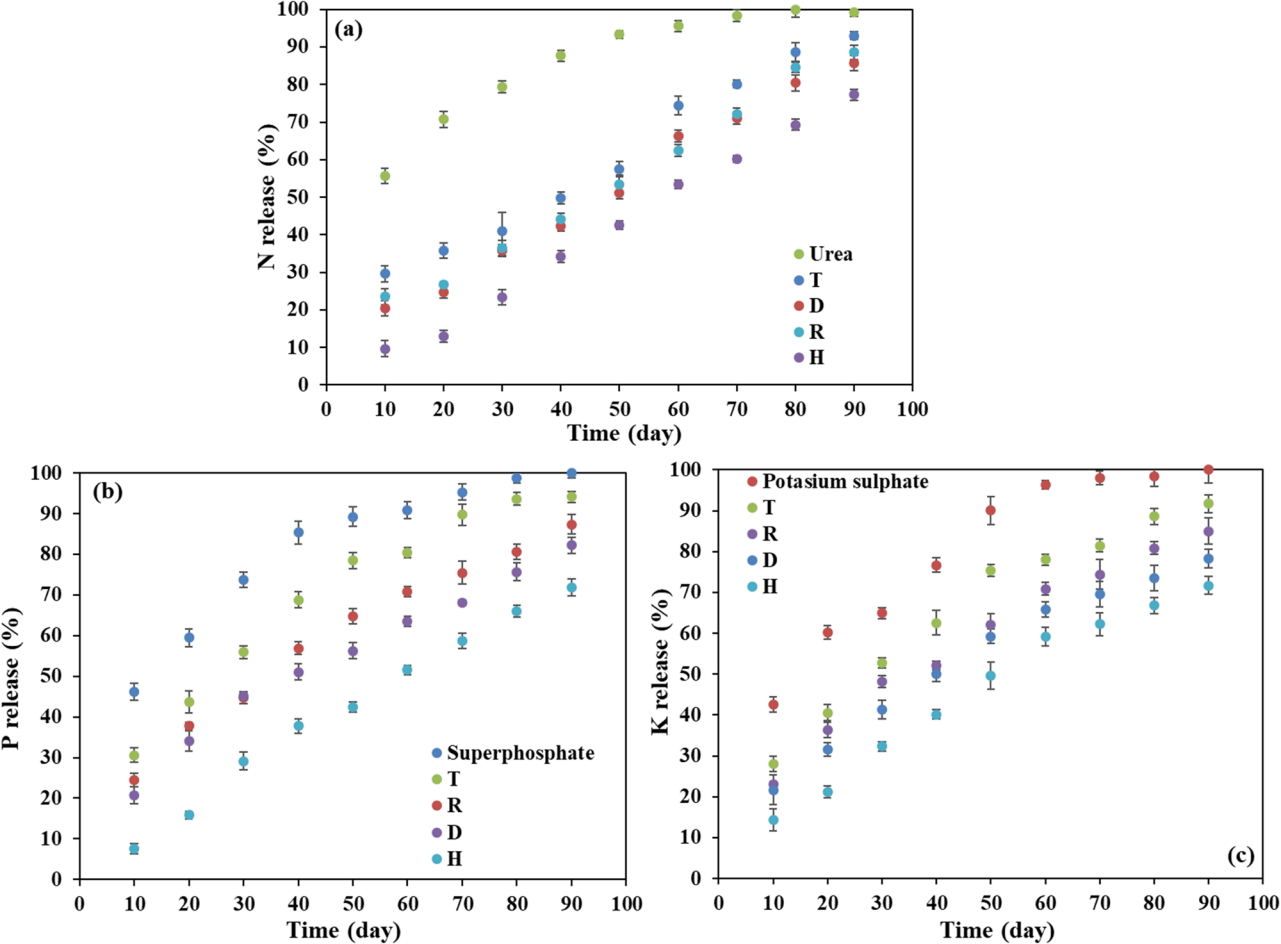
The release percentage of (**a**) N, (**b**) P, and (**c**) K from the synthesized SRFs in the cultivation substrate. Treatments: R (NPK coated with coating formulations), D and H (NPK coated with coating formulations + NPK loaded in nanocomposite hydrogel), NPK (traditional NPK without coating/load), and T (commercial SRF-Seasol-PowerFeed-Tomato & vegetables). Details of each treatment were summarized in Table 4.

Investigating the release pattern of nitrogen, phosphorous and potassium for the as-produced slow-release fertilizers (treatments of D, H and R), common NPK and the commercial SRF (T) in the cultivation substrate showed that the nutrients release rates were at highest for the common NPK; and at lowest for the H treatment (Fig. 1). For instance, after 10 days of application of fertilizers, the nitrogen release rate for T, R, D and H fertilizers were 55.7, 29.6, 23.5, 20.5 and 9.6%, respectively. After 60 days, more than 95% of nitrogen has been released from traditional urea granules, while T, R, D and H formulations revealed for 80.2, 72.1, 72.0 and 60.1% of release. The rate of N release in H fertilizer reached 77% ultimately after 90 days (Fig. 1a). Similar trends have been observed for two other elements of phosphorus and potassium (Fig. 1b, c). The release rate of phosphorus from the single superphosphate fertilizer without coating after 10 days of application was 46.1%, and more than 95% of phosphorus got released after 70 days. The release rate of P in T, R, D and H treatments were 30.6, 24.5, 20.6 and 7.6% on the 10 days and 94.1, 87.4, 81.2 and 71.8%, on the 90 days after application, respectively (Fig. 1b). Potassium released in the rate of 42.5%; 10 days after the application of potassium sulfate without coating, while more than 96% of K got released after 60 days. Potassium release rates in the T, R, D and H fertilizers after 10 days were 27.9, 21.6, 21.6 and 14.3%, respectively, while they reached 91.7, 85.4, 78.3 and 71.7% on the 90 days after application (Fig. 1c). In general, the release rate of elements in synthesized fertilizers were slower than the commercial fertilizers, the rate release in the D and R formulations were similar for all the elements, and when 30 wt.% of coated fertilizers was replaced with nanocomposite hydrogel containing fertilizers (H treatments) the slowest release rate for all nutrient elements was provided. Accordingly, the H treatment compare to other treatments might boost plant production and environmental safety. This could be hypothesized that combination of different strategies in preparation of SRF would be resulting in their synergistic effect particularly if they used in appropriate dosages/ratios. Vudjung et al. developed a biodegradable hydrogel based on pre-vulcanized natural rubber (NR) and cassava starch as a coating membrane for urea^40^. They used the wax as outer coating shell to further improve the release characteristic of the coated urea and showed that NR/St ratios could affect water swelling, water permeability and biodegradation of coating layer. The urea bead (UB) coated with hydrogel and wax layers (W-IPN-CUB) exhibited exceptional release behavior up to 24 days in soil compared to native UB (3 days) and also significantly improved the performances of corn and basil height compared to native UB^40^. In another report, different biocomposites (CF1-3) based on starch acetate (SA)/polyvinyl alcohol (PVA)/Glycerol (GLY) were used with different layers for the control of P and N releases from diammonium phosphate (DAP). The release rate results of P and N nutrients in water showed the time to reach maximum concentration of N release was 2.4 and 3.2 times more than the uncoated DAP, when the DAP is covered with CF-3 (70 wt.% of SA) single-layer and double-layer, respectively^41^.

### The fertilizers effects on plant morphology, root and fruit weight

**Figure 2:**
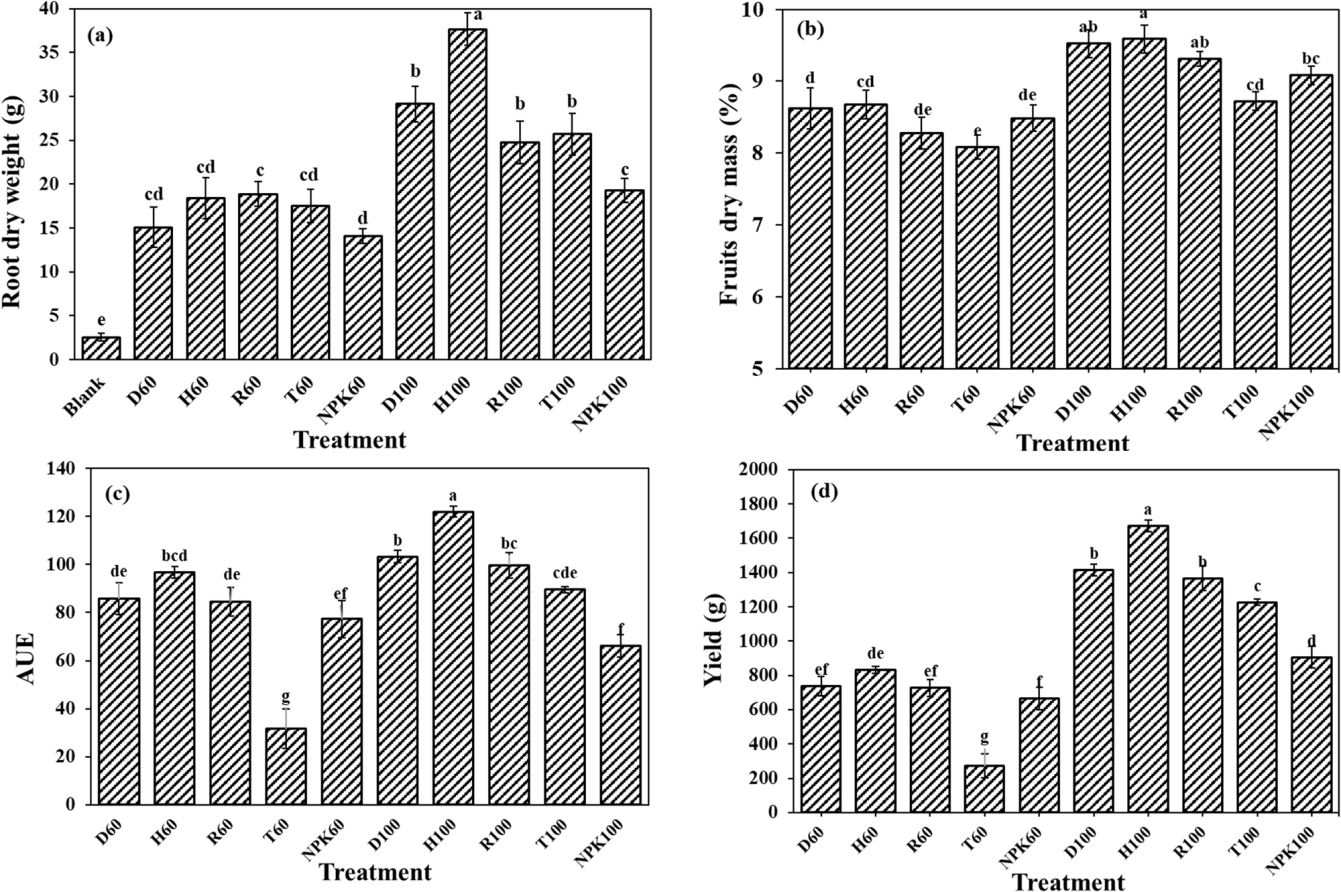
Different fertilizer formulations effects on the (**a**) root dry weight, (**b**) fruits dry mass percentage (**c**) agronomic use efficiency (AUE) of fertilizers, and (**d**) yield in plant, in different fertilizer treatments. Treatments: R (NPK coated with coating formulations), D and H (NPK coated with coating formulations + NPK loaded in nanocomposite hydrogel), NPK (traditional NPK without coating/load), and T (commercial SRF-Seasol-PowerFeed-Tomato & vegetables). Details of each treatment were summarized in Table 3.

Analysis of variance did not show any significant differences between fertilizer treatments affecting the length and diameter of stem and the chlorophyll content. The H100 treatment resulted in the highest value of 37.66 g of root dry weight, which was significantly higher than other fertilizers treatments. The root dry weight increased by 93.22, 59.96, 51.13, 49.88, 53.44, 62.61, 22.64, 34.29, 31.76 and 48.79% by the application of H100, compared to the control, D60, H60, R60, NPK60, D100, R100, T100 and NPK100, respectively (Fig. 2a). Rigas and Chatzoudis revealed that the application of slow-release fertilizers in combination with hydrogel induced increments in yield and dry matter of tomato^42^. Mahgoub also exhibited that the application of hydrogel and chemical fertilizers mixture had significant effect on tomato dry matter^43^. It could be due to the fact that hydrogels can preserve water and nutrients, and consequently help the plant to uptake them more efficiently by their slow release in the rhizosphere^44^. The high solubility of common nitrogen fertilizers, their N loss through leaching, denitrification, and volatilization, will result in N inadequacy for the plant growth and consequently dry matter production^45^. Whereas, slow-release fertilizers application showed the competences to decrease the N leaching and the N conversion to ammonia ratio, they release N accordingly to the plants need, hence are considered as more durable nutrient resource during plant growth period, which could increase dry matter production and yield^43^.

The highest produced fruit dry mass (9.52%) observed in H100 treatment, which compared to T100 and NPK100 treatments showed increments of 9.13 and 5.41%, respectively, even though showed no significant differences with D100 and R100 treatments (Fig. 2b). Similarly, in treatment of 60% fertilizer, the highest dry mass in fruits as for 8.68% obtained in H60 which had no significant differences with D60 and R60, but was 6.91 and 5.41% significantly higher than T60 and NPK60, respectively. Similar findings, i.e., significant effects of the ratios of utilized coating materials for SRF preparation on the crop biomass production have also been reported by other researchers. Beig et al. investigated the effects of different ratios of organic and inorganic blends as coating of urea prills provided by fluidized bed on the spinach productivity^46^. They studied the usage of starch, gelatin, and polyvinyl alcohol in combination with sulfur and plaster of Paris as urea coating substances, along with the paraffin wax and molasses as the binding agents with four different combining weight ratios (C-1 to C-4 treatments). All the formulations along with uncoated urea, and the control (no N: untreated) were applied to spinach crop in the pot experiment. The formulation C-1 yielded the highest urea-N release efficiency and spinach N uptake of 6.87%, and 1.93 g N/pot, respectively, which was four times higher than other coated fertilizers. Coating of urea enhanced spinach fresh biomass/yield between 10–28% after application than uncoated urea^46^. Moreover, previous reports similarly showed the increase in tomato dry/fresh mass with the use of SRF samples. Kinoshita et al. reported the higher tomato fruits dry mass treated with slow-release fertilizers treatment compare to liquid fertilizer (commonly used in a soilless culture), and with increasing the amount of applied slow-release fertilizer, the dry mass in fruits was increased^47^. In another study, controlled-release urea (CRU) samples were synthesized by a biobased nanocomposite of lignin and bentonite as a coating material for urea. Five types of CRU were prepared using different ratios of modified bentonite and their efficiency in improving the growth of tomato was studied under field conditions, at three N levels. The results showed that all CRU treatments at the three N levels significantly enhanced plant fresh weight, and dry weight, compared to the control^48^.

### The agronomic use efficiency of fertilizers and their effects on the yield production

The highest agronomic use efficiency of fertilizers observed in H100 treatment in amount of 122.01, which was higher than NPK100, T100, R100, and D100 treatments respectively for 45.85, 26.60, 18.22, and 15.29%, respectively (Fig. 2c). The improved recovery of 45% over the NPK fertilizers, defined the importance of these formulations in reducing the chances for nutrients leaching, environment pollution and costs. Regarding the fact that the highest recovery has been observed in the fertilizer containing hydrogel, it seems that the application of hydrogels containing chemical fertilizers render lower rate of releasing nutrients which heighten their retention in the soil, improve soil quality and fertilizer use efficiency^9^. The production yield showed similar trend as AUE among the fertilizer treatments (Fig. 2d). The highest production yield in plant treated with H100 fertilizer was 1671.54 g, which had shown significant differences with other fertilizers treatments, more specifically with NPK100, T100, and then with R100 and D100 (Fig. 2d). Mahgoub revealed that the application of hydrogel along with only a half of the fertilizer requirement, increased the yield in tomato and the possible reasons for that would be the higher availabilities for water and nutrients, and promoting their uptake by plants^43^. In accordance with our results, Helal et al. found that the marketable yield and the total tomato fruit yield/plant were affected significantly by controlled-release urea (CRU) treatments and they were significantly higher than those for the control (902.55 vs. 418.06 g/plant)^48^. Li et al. investigated the effects of two slow-release nitrogen fertilizers and irrigation on the yield of greenhouse tomato. They used two slow-release nitrogen fertilizers (polymer-coated urea (PU) and carbon-based urea (CU). Compared with urea (U application), the CU application increased tomato yield by 4600 kg ha^−1^ and net income from tomato cultivation by 6313 yuan/ha^49^.

### The fertilizers effects on the nutrient concentrations remained in the cultivation substrate

**Figure 3:**
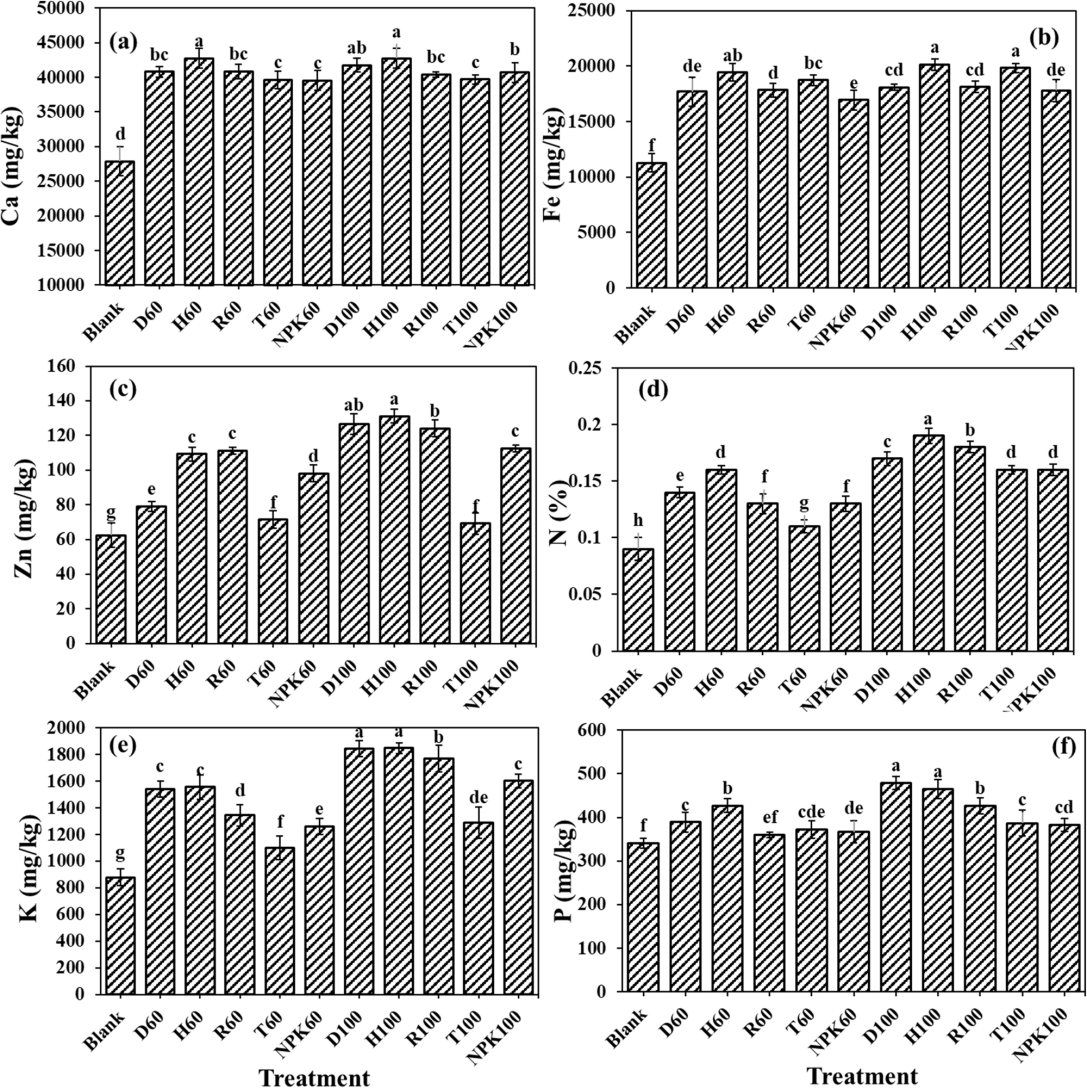
The fertilizers effects on the (**a**) calcium (Ca), (**b**) iron (Fe), (**c**) zinc (Zn), (**d**) nitrogen (N), (**e**) potassium (K), and (**f**) phosphorus (P) concentrations remained in the cultivation substrate. Treatments: R (NPK coated with coating formulations), D and H (NPK coated with coating formulations + NPK loaded in nanocomposite hydrogel), NPK (traditional NPK without coating/load), and T (commercial SRF-Seasol-PowerFeed-Tomato & vegetables). Details of each treatment were summarized in Table 3.

The investigation on the remained elements in the cultivation substrate revealed that the highest remained amounts of elements including calcium, iron and zinc were respectively 42,731.71 mg kg^−1^, 20,137.93 mg kg^−1^ and 131.03 mg kg^−1^, in pots treated with H100 fertilizer (Fig. 3a–c). Calcium concentration in this treatment showed significant difference with the other fertilizers treatments, except for H60 and D100 formulations. The increment percentage of the remained Ca in H100 treatment, compare to control, NPK100, and T100 were 34.76, 4.77 and 7.11%, respectively. The amount of the remained iron in the cultivation substrate treated with H100, with the exception of H60 and T100, showed significant differences with other fertilizer treatments as it increased the remained amount for 43.96, 11.61 and 10.02%, respectively, compared to the control, NPK100 and R100 treatments. The comparison for the remained zinc concentration in the substrate showed the highest amount in H100, which did not show significant difference with D100 but with other fertilizers treatments. The remained zinc concentration in pots treated with H100 was 52.39, 14.08, 47.26 and 5.30% significantly higher than control, NPK100, T100 and R100 treatments, respectively. Similarly, the highest nitrogen percentage of 0.19 in the H100 treatment observed, which were significantly higher than control, NPK100, T100, R100, and D100 treatments, respectively, for 52.63, 15.79, 5.26, 5.26 and 10.525 (Fig. 3d). The highest K remained in the substrate 1848.26 mg kg^−1^ was seen in pots treated with the H100 fertilizer, which was higher than control, NPK100, T100, and R100 treatments by 52.50, 13.38, 30.28, and 4.42% (Fig. 3e). Nonetheless, the highest amount of phosphorus remained in the substrate has been observed in pots treated with D100 fertilizer treatment which showed no significant difference with the H100 treatment, but it was significantly higher than control, NPK100, T100 and R100 treatments, respectively, for about 28.93, 20.08, 19.31 and 10.97% (Fig. 3f). Similar results were reported by Song et al. that evaluated the effects of commercially available fertilizers compared to the two different SRFs (prepared by physical mixing and chemical reaction of NPK-containing polymer slow-release fertilizer and hydroxyethyl cellulose water-absorbing resin) on tomato in pot experiment. Their results displayed that the soil nutrient content and enzyme activity increased compared with the commercially available fertilizer control soil, and the ammonium nitrogen and nitrate nitrogen content increased by 16.61%, 48.06%, and 21.61%, 84.64%, respectively^50^. Rop et al. studied the effects of formulated nano-NPK SRF composite on the performance and yield of three plants of maize, kale and capsicum. Their findings revealed that at final harvest, soil samples planted with maize and amended with SRF had significantly higher P content, compared to the control^51^. It could be hypothesized that the application of entrapped fertilizer in hydrogel along with the coated fertilizer not only increased the time in which water and nutrients remain in the substrate for plant use, but also eliminated the chances for any loss by incorporation the nutrients in the hydrogel structure. Thereby the slow release of nutrients from these synthesized fertilizers would extend the accessibility to the nutrients for plants during growth period. Wang et al. showed that the application of slow-release fertilizers increased the total and available concentrations of N, P and K in the soil. Hydrogel application reduced the micro-nutrients leaching but increased the water use efficiency^52^. Reducing the chances of nutrients leaching from the soil by the application of hydrogel would be resulted in lowering the fertilizer application^53^, and increasing the sustainability in the system.

### The fertilizers effects on the nutrient accumulation in the aboveground biomass

**Figure 4:**
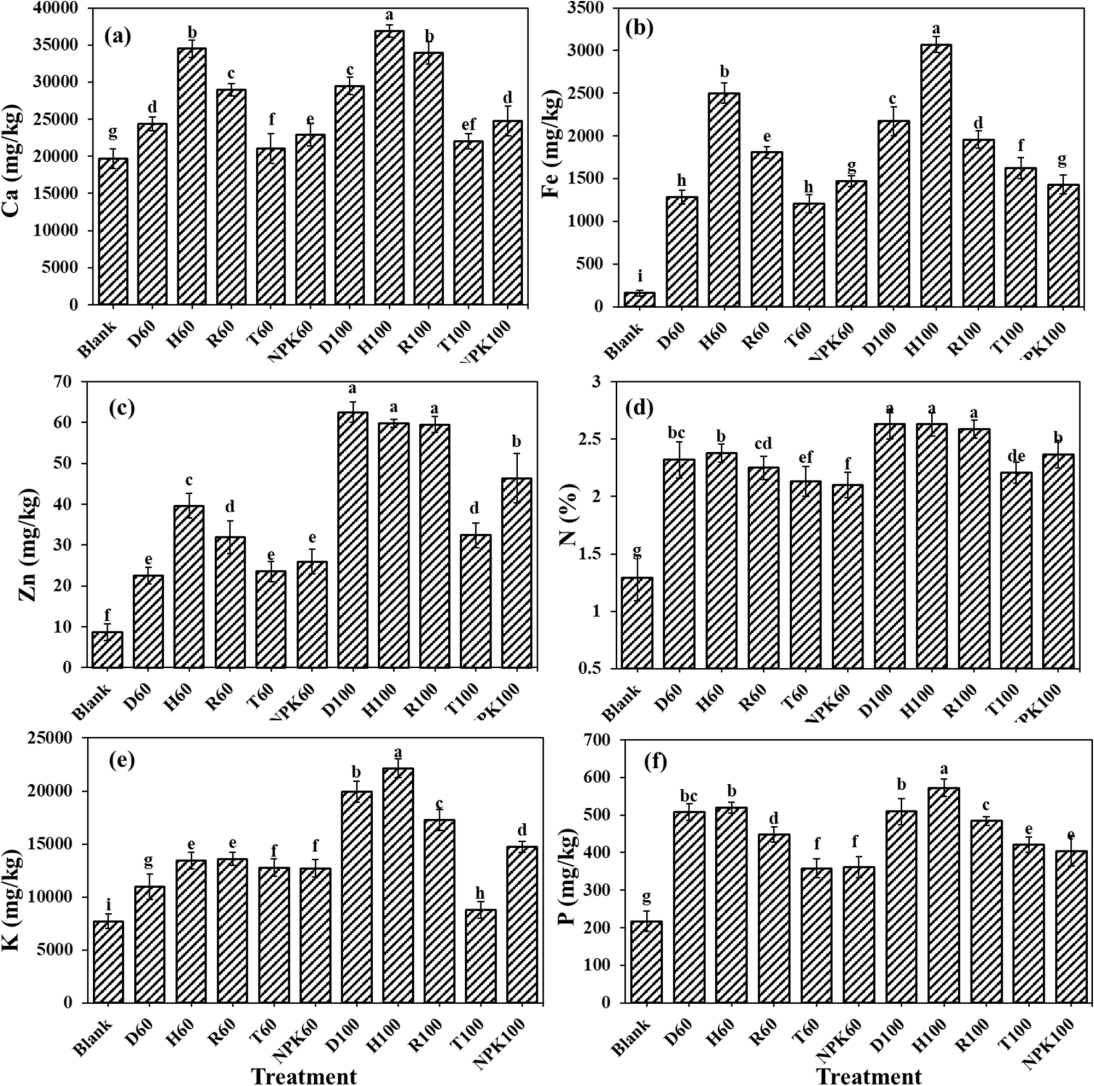
The fertilizers effects on the (**a**) calcium (Ca), (**b**) iron (Fe), (**c**) zinc (Zn), (**d**) nitrogen (N), (**e**) potassium (K), and (**f**) phosphorus (P) concentrations in the aboveground biomass. Treatments: R (NPK coated with coating formulations), D and H (NPK coated with coating formulations + NPK loaded in nanocomposite hydrogel), NPK (traditional NPK without coating/load), and T (commercial SRF-Seasol-PowerFeed-Tomato & vegetables). Details of each treatment were summarized in Table 3.

The highest amounts of iron and zinc as micro-elements were observed in H100 and D100, respectively, which both contained hydrogel. This result indicates the hydrogel role in preserving nutrients for longer time in the cultivation substrate. The H100 fertilizer application induced the highest calcium concentration in the aboveground biomass in amount of 36,892 mg kg^−1^, which was significantly higher compare to other fertilizer treatments (Fig. 4a). The amount of calcium in aboveground treated by H100 showed 48.75, 46.66, 40.27, 20.04, and 8.0% increments compared to NPK100, control, T100, D100, and R100, respectively. The highest amount of iron in the aboveground biomass was 3070.63 mg kg^−1^, which was 94.73, 53.46, 47.16, 36.28, and 9.25% higher than control, NPK100, T100, D100, and R100 treatments, respectively (Fig. 4b). Moreover, the highest amount of zinc was 62.5 mg kg^−1^ which was higher than control, T100 and NPK100 around 86.09, 48.15, and 25.82, respectively (Fig. 4c). The highest N concentration was 2.36% in both H100 and D100 treatments, which was 50.95, 15.96, and 9.88 percent higher than control, T100 and NPK100 treatments (Fig. 4d). Rigas and Chatzoudis revealed that the application of hydrogel along with slow-release fertilizers stimulated higher nitrogen adsorption in tomato plants^42^. The highest potassium concentration in aboveground biomass treated with H100 fertilizer was 22,148.51 mg kg^−1^, which was 65.26, 60.38, 33.47, 21.96, 9.93% significantly higher compared to control, T100, NPK100, R100, and D100, respectively (Fig. 4e). Finally, the highest amount of phosphorus in aboveground biomass of 572.12 mg kg^−1^ observed in pots treated with H100 which was 62.05, 29.43, 26.49, 15.37, and 10.98% higher than control, NPK100, T100, R100, and D100, respectively (Fig. 4f). Joner et al. reported that the application of hydrogel stimulated the enzyme activity in the soil such as phosphatase which would increase phosphorus availability for plants^54^.

### The fertilizers effects on the nutrient adsorption by roots

**Figure 5:**
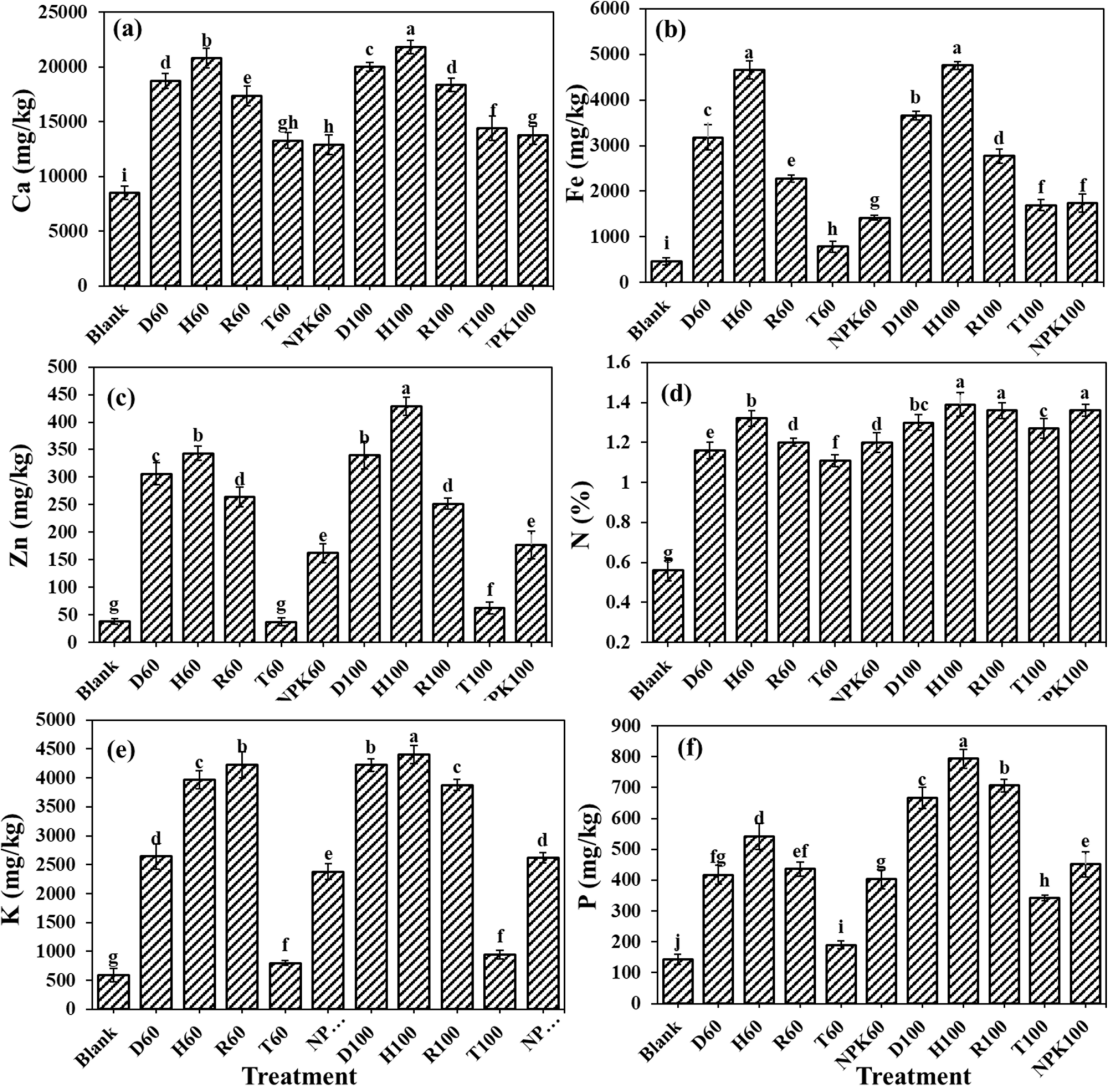
The fertilizers effects on the (**a**) calcium (Ca), (**b**) iron (Fe), (**c**) zinc (Zn), (**d**) nitrogen (N), (**e**) potassium (K), and (**f**) phosphorus (P) concentrations in root biomass. Treatments: R (NPK coated with coating formulations), D and H (NPK coated with coating formulations + NPK loaded in nanocomposite hydrogel), NPK (traditional NPK without coating/load), and T (commercial SRF-Seasol-PowerFeed-Tomato & vegetables). Details of each treatment were summarized in Table 3.

Results showed that H100 fertilizer treatment induced the highest amounts of iron, zinc and calcium adsorbed by tomato roots. The highest amount of calcium was 21,823.81 mg kg^−1^, which was higher than control, NPK100, T100, R100, and D100 treatments for about 61.12, 36.95, 34.06, 16.06, and 8.37%, respectively (Fig. 5a). The highest iron concentration in roots were 4757.14 mg kg^−1^ which was 90.19, 64.34, 63.55, 41.80, and 23.03% higher than control, T100, NPK100, R100, and D100, respectively (Fig. 5b). Additionally, the greatest zinc concentration in roots of tomato treated with H100 was 428.57 mg kg^−1^ which was higher than control, T100, NPK100, R100, and D100 treatments, respectively, for 91.14, 85.41, 58.56, 41.20, and 20.75% (Fig. 5c). Zinc uptake by plants could be done through both active and inactive pathways, among different environmental characteristics; temperature and aeration are the most important factors that could influence the active zinc uptake in plant^55^. Thereby the application of hydrogel nano-composites which improves the aeration in the rhizosphere could stimulate more active uptake for zinc by the roots^56^. The highest concentrations of nitrogen, phosphorus and potassium in roots were seen in the pots treated with H100 treatment as well, respectively in amounts of 1.39%, 666.66 and 4221.69 mg kg^−1^ (Fig. 5d–f). Nitrogen concentration in H100 treatment was respectively 59.71, 8.63, 6.47% higher than control, T100 and D100; but showed no significant differences with R100 and NPK100 treatments. Potassium concentration in roots treated with H100 treatment was 86.57, 78.41, 40.56 11.92, and 4.15% higher than control, T100, NPK100, R100, and D100, respectively. In addition, Phosphorus concentration in H100 treatment was 82.01, 57.04, 43.17, 16.04 and 10.98% higher than control, T100, NPK100, R100, and D100, respectively. Nassaj et al. exhibited that the application of hydrogel nano-composites induced higher potassium concentration in tomato roots, since hydrogel promotes the optimum conditions regarding to the aeration and moisture in the soil^45^. Mahgoub revealed that hydrogel application with the 50% of fertilizer requirement, increased the nitrogen, phosphorus and potassium uptake by tomato plants^43^. In summary, hydrogels through preserving soil moisture and diminishing the possibility for nutrients leaching could indirectly affect the nutrients uptake in plants^57^.

### The fertilizers effects on nutrient concentrations in the fruits

**Figure 6:**
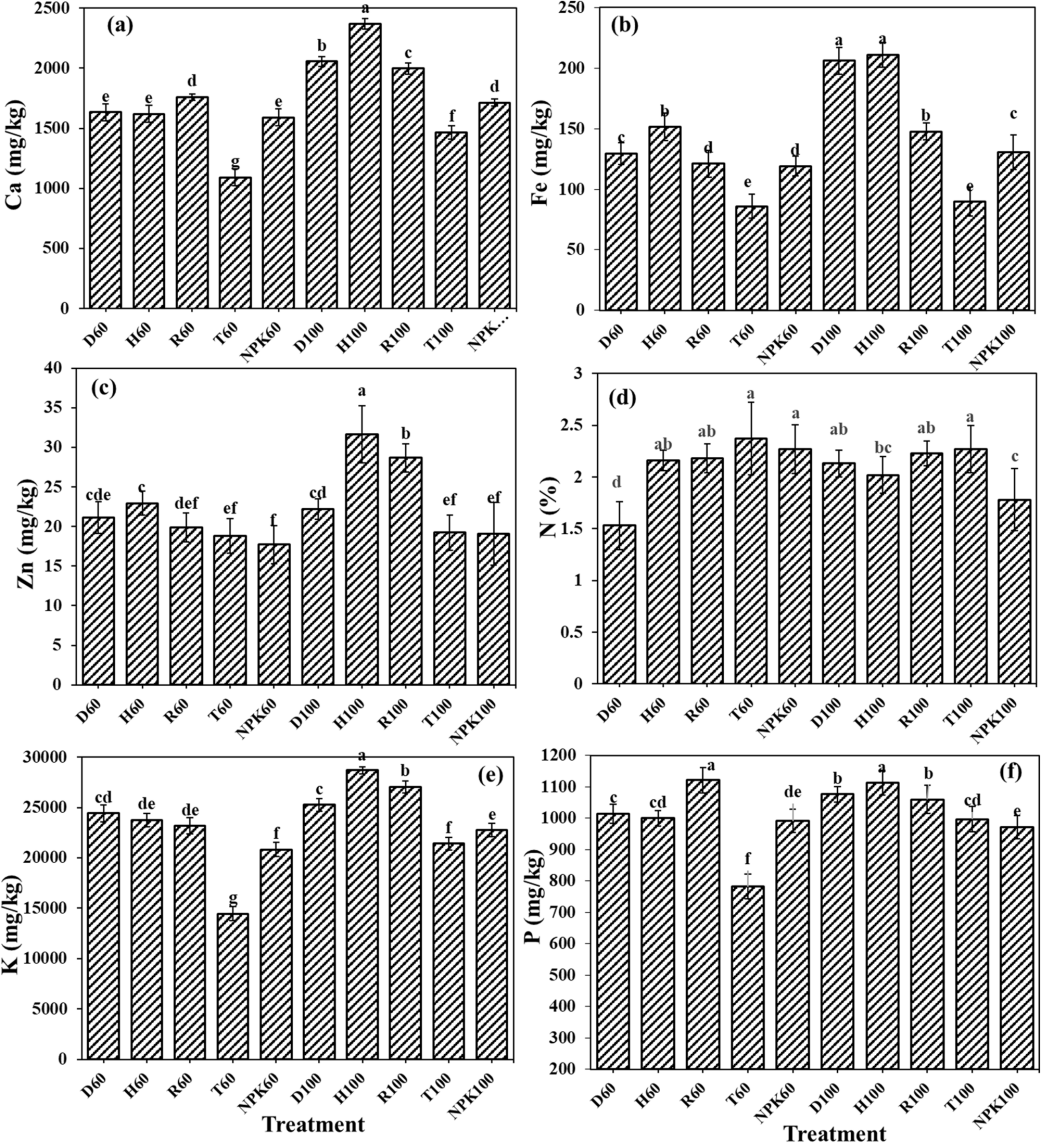
The fertilizers effects on nutrients concentration (**a**) calcium (Ca), (**b**) iron (Fe), (**c**) zinc (Zn), (**d**) nitrogen (N), (**e**) potassium (K), and (**f**) phosphorus (P) concentrations in tomato fruits. Treatments: R (NPK coated with coating formulations), D and H (NPK coated with coating formulations + NPK loaded in nanocomposite hydrogel), NPK (traditional NPK without coating/load), and T (commercial SRF-Seasol-PowerFeed-Tomato & vegetables). Details of each treatment were summarized in Table 3.

Analysis of variance showed the highest concentrations for Ca (2369.72 mg kg^−1^), Fe (211.01 mg kg^−1^), and Zn (31.62 mg kg^−1^) in fruits of plants treated by H100 treatment (Fig. 6a–c). Calcium concentration in H100 treatment compare to T100, NPK100, R100, and D100 treatments was 38.18, 27.68, 15.73, and 13.27% higher, respectively. While iron concentrations in H100 and D100 were not significantly different but it was 57.46, 38.02, and 30.04% higher than T100, NPK100, and R100 treatments, respectively. Zinc concentration in H100 treatment was also higher than NPK100, T100, D100, and R100 treatments for 39.75, 39.17, 29.71, and 9.34%, respectively. Sita et al. showed that hydrogel application increased more growth, calcium and magnesium uptake in *Dendrathema grandiflorum*^58^. The highest nitrogen uptake (2.37%) observed in T60 treatment, with 35.44, 24.89, and 14.76% higher significant value compared to D60, NPK100, and H100 treatments, respectively (Fig. 6d). Considering the fact that the highest nitrogen adsorption in aboveground biomass was observed in H100 treatment and taking into account that fruits in this treatment were in larger size in comparison to other treatment probably dilution in total mass of fruit was the reason for the lower N concentration in fruits of H100 treatment compared to others. The highest concentration for potassium (28,675.16 mg kg^−1^) adsorbed in fruits of plants treated with H100 treatment while it showed 25.32, 20.67 and 11.95, and 5.70, % higher amounts compared to T100, NPK100, D100, and R100 treatments (Fig. 6e). Similarly, the highest phosphorus concentration (1112.68 mg kg^−1^) was seen in H100 treatment, which showed no significant difference with T60 but showed higher significant percentage value compared to NPK100, T100, R100, and D100 for 12.69, 10.41, 4.76, 3.26%, respectively (Fig. 6f). In general, understanding the relationship between releasing rates of macro-nutrients including N, P, and K on the uptake of other nutrients like Ca, Zn, and Fe requires more investigations. While the double interactions between macro- and microelements have been known in plants at the physiological level, but the molecular mechanisms and signaling pathways which justified such cross-talks in plants were not recognized properly, yet^59^. In the most literature published, the effects of release rate on macro-nutrients uptake (NPK) were evaluated, and to the best of our knowledge, there was no report on the effect of release rate on micro-nutrients and calcium, their availability and uptake. However, according to the results of current study, the H100 treatment which contained both coated fertilizers with polymer latex and entrapped fertilizers in the hydrogel, guaranteed the best results due to slow release of the chemical fertilizer and increasing water and nutrients holding capacity in the rhizosphere. In that case, nutrients could get adsorbed by plants slowly throughout growth period as they need with less energy consumption^60^. Additionally, these polymeric compounds induce more roots in plants, which increased plant ability for more water and nutrients uptake and reduced the chances of any possible stressful condition such as nutrients deficiency and preserved the ionic homeostasis in plants, which accordingly stimulated more micro-nutrients uptake^59^.

### The fertilizers effects on lycopene, antioxidant capacity and vitamin C in the fruits

**Figure 7:**
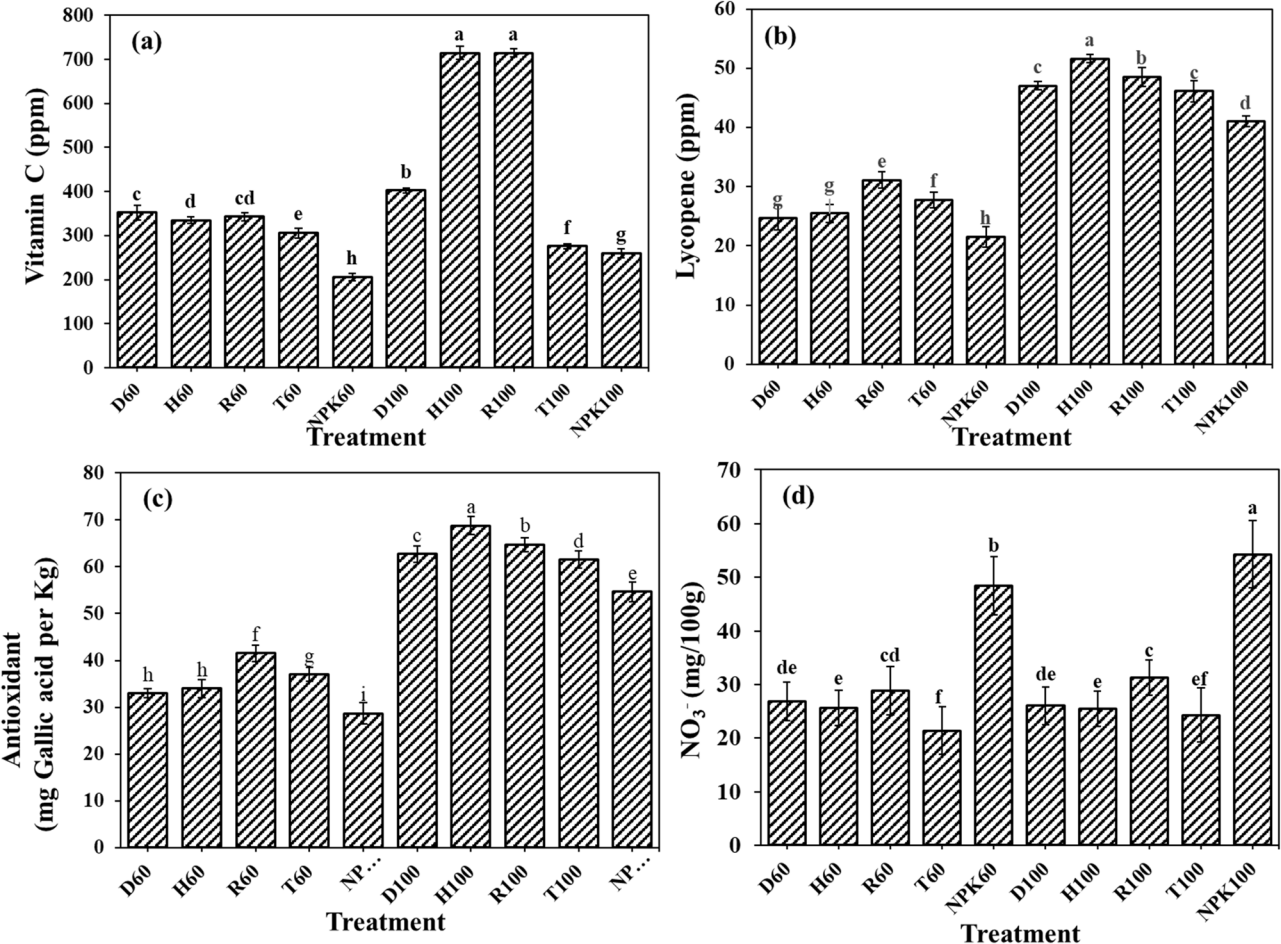
The fertilizers effects on the (**a**) vitamin C, (**b**) lycopene concentration, and (**c**) antioxidant capacity in tomato fruits. (**d**) Nitrate accumulation in tomato fruits affected by different fertilizer treatments. Treatments: R (NPK coated with coating formulations), D and H (NPK coated with coating formulations + NPK loaded in nanocomposite hydrogel), NPK (traditional NPK without coating/load), and T (commercial SRF-Seasol-PowerFeed-Tomato & vegetables). Details of each treatment were summarized in Table 3.

The highest amount of vitamin C (714.3 mg L^−1^) observed in pots treated with H100 and R100, which was 63.63, 61.40, and 43.60% higher than NPK100, T100, and D100 treatments (Fig. 7a). Lycopene recognized as a natural antioxidant with anti-carcinogenic properties which is responsible for the red color in the tomato fruits and has high economic importance^61,62^. Hence, lycopene concentration in tomato played important economical and nutritional role. Lycopene concentration was seen in the highest amount of 51.6 mg L^−1^ in plants affected by H100 treatment, which in compare with the other treatments with same amounts of fertilizers including NPK100, T100, D100, and R100, was respectively higher about 20.52, 10.62, and 8.82, and 6.0% (Fig. 7b). The H100 treatment additionally induced the highest antioxidant capacity as for gallic acid in tomato plants in amount of 68.8 mg kg^−1^, which in compare to NPK100, T100, D100, and R100, respectively, was higher for about 20.4, 10.5, 8.8, and 5.9% (Fig. 7c). Gallic acid as a phenolic compound is effective in plant defense against pathogens, which besides its antioxidant property inhibits auxin oxidation, thereby auxin aggregation promotes root growth^63^. Similarly, earlier studies revealed that hydrogel application induced higher concentrations of ascorbic acid and lycopene in tomato^64,65^. Application of two slow-release nitrogen fertilizers (polymer-coated urea (PU) and carbon-based urea (CU) for greenhouse tomato production resulted in increased soluble sugar, vitamin C, and lycopene contents of fruits. For instance, lycopene content in tomato in native urea treatment were 6.95 µg/g, while it reached to 9.25 and 8.95 µg/g in CU and PU treatments, respectively^49^. Therefore, the application of SRF formulations could improve tomato quality which might have rooted from the slow release of nutrients that helps prevention of the rapid/excessive absorption of nutrients to ensure efficient nutrient conversion by plants^49^.

### The fertilizers effects on the nitrate accumulation in tomato fruits

As depicted in the Fig. 7d, the highest nitrate concentration in tomato fruits was detected in the NPK100 treatment, which was around more than 50% of nitrate concentration affected by other 100% treatments. Similarly, in the case of 60% fertilizer treatments, NPK60 treatment showed the highest amount of accumulated nitrate which was higher in range of 55.78, 47.10, 44.42, and 40.95% compared to T60, H60, D60, and R60 treatments, respectively. These obtained results have been expected to found according to the results of nutrients release experiments. As Fig. 1 showed the highest nutrients release including N, P, and K were observed in NPK treatments (both 60 and 100% of recommended rates), which provided the immediate source of nutrients for plants. Following uptake of large amount of nitrate in a short period of time by the application of NPK fertilizers, plants had not enough time for nitrate reduction to ammonia, hence most of nitrate got accumulated in plants. A reduction in nitrate accumulation by the application of slow-release fertilizers probably is due to slow releasing nitrogen because of the existence of nano particles in the coating structure of these fertilizers along with hydrogel entrapment. Wang et al. revealed that nitrate reductase enzyme activity, which is essential for nitrate reduction to ammonia, has been decreased in the leaf of Chinese Chives treated with common fertilizer treatment compared to the slow-release fertilizer^52^. Li et al. showed with application of polymer-coated urea (PU) and carbon-based urea (CU) as SRFs for tomato the nitrate content in fruits were significantly reduced compared to uncoated urea application. While the nitrate content of fruit in urea were found to be 339 mg kg^−1^, the PU and CU revealed nitrate content of 300 and 311 mg  kg^−1^, corresponding to 11% and 8% reduction, respectively^49^. The high concentration of nitrogen released and accumulated in plants in a short period of time disrupted the balanced metabolism for carbon and nitrogen and thereby restrained nitrate reductase enzyme activity^45^. The slow-release fertilizers release the nutrients in relation with plant needs hence reduce the excessive fertilization and nutrient leaching and provide the root system with enough nutrients which increase root activity^52^. Ye et al., explained that if sufficient nitrogen were available for plants, plant growth and production are guaranteed, while the excessive supply of nitrogen induced nitrate accumulation, and the adverse effects on human health^66^.

## Conclusion

This study described synthesis and application of three water-based bio-polymeric formulations in preparation of the advanced and efficient slow release NPK fertilizers. To this goal, starch-*g*-poly(styrene-*co*-butylacrylate) waterborne latex and carnauba wax emulsion were used for coating the urea, potassium sulfate, and superphosphate fertilizer granules. Besides, starch-*g*-poly (acrylic acid-*co*-acrylamide) nanocomposite hydrogel was used for entrapment of the aforesaid fertilizers. Combination of the coated and entrapped fertilizers at different ratios resulted in three different SRF samples that their efficiencies has been confirmed in greenhouse trial on tomato production compared to the traditional NPK fertilizer and a commercial SRF sample. Application of such combined natural-based SRF samples could provide numerous advantages including: (1) utilization of starch and carnauba wax as natural, renewable and biocompatible resource candidates rather than non-biodegradable synthetic polymers; (2) employment of NCNPs as feasible and natural-based nano-filler for reinforcement of polymer formulations to improve nutrient release profile; (3) water-based characteristic of the coating formulations to provide advantages of nontoxicity, economy, and efficiency compared to the conventional organic solvent-based formulations; (4) utilization of one-pot and scalable synthesis procedure for preparation of SRF samples; (5) Tailoring the ratios of nutrient components and fertilizer longevity using altering the feed nutrients, ratios of nutrient to coating formulations and number of coating layers. Finally, development of such green, efficient and innovative SRF samples could practically tackle the global challenge for increasing the crop yield and food quality with the minimum possible ecological footprint.

## Data Availability

The datasets used and/or analyzed during the current study available from the corresponding author on reasonable request.
